# Physically active adults with uncomplicated type 1 diabetes exhibit normal cardiopulmonary exercise test responses versus healthy controls

**DOI:** 10.14814/phy2.70997

**Published:** 2026-06-30

**Authors:** Samu Sorola, Timo Eronen, Vesa Hyrylä, Saana Kupari, Mika Venojärvi, Heikki Tikkanen, Mika Tarvainen, Harri Lindholm

**Affiliations:** ^1^ Institute of Biomedicine University of Eastern Finland Kuopio Finland; ^2^ Department of Technical Physics University of Eastern Finland Kuopio Finland

**Keywords:** cardiopulmonary exercise test, physical activity, uncomplicated type 1 diabetes

## Abstract

We investigated whether physically active adults with uncomplicated type 1 diabetes mellitus (T1DM) differ from healthy controls in cardiopulmonary responses during incremental cardiopulmonary exercise testing (CPET). Fifteen adults with uncomplicated T1DM (duration 15 ± 7 years) and 33 matched controls (age, sex, height, and BMI) performed an incremental cycling CPET and self‐reported their weekly physical‐activity levels. Pulmonary and cardiovascular variables were recorded at rest, ventilatory thresholds 1 and 2, and peak effort, with noninvasive cardiac function assessed by impedance cardiography. Weekly light‐intensity activity frequency differed between groups according to the Mann–Whitney *U* test (*p* = 0.028), indicating a statistically significant distributional difference. However, the Hodges–Lehmann estimated median difference was not statistically significant because the 95% CI included zero (0–3). No other self‐reported physical activity variables differed between the groups. In the linear mixed‐effects models, no significant Group effects or Group × Stage interactions were observed for any cardiopulmonary variable during CPET. In this physically active and clinically selected cohort, adults with uncomplicated T1DM showed no statistically detectable differences in cardiopulmonary responses compared with healthy controls under the present CPET conditions. Larger studies are needed to confirm these findings and evaluate potential subclinical hemodynamic or autonomic differences.

## Graphical abstract

Physically active adults with uncomplicated type 1 diabetes and healthy controls completed incremental cycling cardiopulmonary exercise testing with measurements at rest, ventilatory thresholds 1 and 2, and peak exercise. Linear mixed‐effects models showed no statistically detectable Group effects or Group × Stage interactions for conventional cardiopulmonary variables under the present CPET conditions.

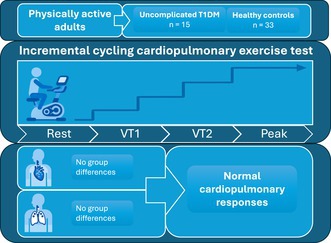

## 1 INTRODUCTION

Cardiorespiratory fitness plays a pivotal role in preventing cardiovascular disease (CVD) progression in individuals with type 1 diabetes mellitus (T1DM) (Wu et al., 2019). A deeper understanding of the mechanisms underlying acute exercise responses in uncomplicated cases may yield insights into early cardiovascular alterations associated with diabetes. Therefore, this study focuses on adults with uncomplicated T1DM to minimize confounding from diabetes‐related complications.

T1DM is characterized by the destruction of pancreatic beta cells by the immune system, resulting in insulin therapy dependency. Cardiovascular risks remain high, and CVDs are a leading cause of mortality in T1DM, even with good metabolic control (Colom et al., 2021). Globally, the prevalence of T1DM and its societal burden are increasing rapidly (Gregory et al., 2022; You & Henneberg, 2016), while CVD prevention strategies in T1DM are often based on type 2 diabetes mellitus studies, despite the underlying pathophysiological mechanisms of these two conditions differing substantially (Colom et al., 2021). Therefore, direct application of T2DM‐based evidence to T1DM populations may not always be appropriate, highlighting the need for T1DM‐specific research. Hence, it is critical to explore the impact of T1DM on cardiopulmonary function during exercise in individuals with no diagnosed diabetes‐related microvascular complications (neuropathy, nephropathy, and retinopathy), hypertension, or any chronic diseases except for T1DM (i.e., uncomplicated T1DM).

Cardiopulmonary exercise testing (CPET) measures the body's multiorgan response to exercise, serving as a tool to pinpoint cardiovascular, pulmonary, muscular, or combined issues. The first and second ventilatory thresholds (VT1 and VT2) are physiological markers that reflect the body's response to increasing exercise intensity. VT1 marks the onset of lactate accumulation, where ventilation (V̇E) begins to rise to manage the increasing production of carbon dioxide, while VT2 indicates a more rapid accumulation of lactate, leading to a significant increase in ventilation as the body approaches its anaerobic limits in both normally healthy individuals and individuals with T1DM (Gaskill et al., 2001; Moser et al., 2018).

T1DM has been shown to have lower performance at VT1 and VT2, likely due to altered metabolism (Eckstein et al., 2021). A typical response to CPET in long‐term T1DM is attenuated maximal oxygen uptake (V̇O_2_max) (Eckstein et al., 2021; Koponen et al., 2013; Wilson et al., 2017). The heart rate (HR) to performance curve is also abnormal: instead of the normal inverse curve, where HR rises sharply at low workloads and then flattens toward peak effort in recreationally active adults, people with T1DM show an almost linear curve. This linear curve, which is accompanied by delayed stroke volume (SV) response to incremental exercise, may reflect diabetes‐related changes in myocardial function (Haennel et al., 1993). Similar findings have been observed in T1DM individuals without long‐term complications (Moser et al., 2018). In uncomplicated T1DM, while respiratory function appears normal at rest, CPET has revealed blunted responses at peak intensities, including V̇O_2_max (Jlali et al., 2023; Peltonen et al., 2012). However, Wilson et al. (2017) found that although V̇O_2_max tends to be lower during CPET, it is not statistically significant, and Hyrylä et al. (2022) found that T1DM exhibited attenuated expiratory flow at peak exercise independent of peak oxygen uptake and minute ventilation. Long‐term glycemic control may contribute to aerobic capacity in T1DM (Poortmans et al., 1986), although glycated hemoglobin A_1c_ (HbA_1c_) alone does not fully explain the heterogeneity of CPET responses (Eckstein et al., 2022).

Therefore, the present study focused on physically active adults with uncomplicated T1DM to minimize confounding from diagnosed diabetes‐related complications, hypertension, cardiovascular medication, smoking, and major comorbidities. This selection was intentional, as the aim was not to characterize CPET responses in the broader heterogeneous T1DM population, but to examine whether uncomplicated T1DM itself was associated with altered conventional CPET responses under controlled study conditions. We hypothesize that individuals with uncomplicated T1DM may still exhibit subclinical signs of pulmonary and cardiac dysfunction during incremental exercise. Identifying these subclinical alterations could deepen our understanding of early T1DM progression and clarify how it interacts with cardiorespiratory fitness and overall health. Thereby, we investigated cardiopulmonary responses at four specific time points of the CPET protocol, that is at rest, at ventilatory thresholds (VT1 and VT2), and during peak effort.

## 2 METHODS

The data used in this study originated from the Effects of Exercise and Stress on Glucose Metabolism in Type 1 Diabetes (DIAMES) project, conducted at the University of Eastern Finland between 2021 and 2022. The DIAMES study received ethical approval from the ethics committee of the Northern Savo Hospital District, Kuopio, Finland (reference number 409/2019). The study adhered to the principles of the latest version of the Helsinki Declaration, good clinical practice, and the General Data Protection Regulation (GDPR). All participants provided written informed consent before participating in the study, and no financial compensation was offered for their involvement.

### 2.1 Participants

A total of 53 participants (T1DM = 18, control = 35) completed the study. Of these, three T1DM participants were excluded due to hypertension medication, and two control group participants due to significant electrocardiography (ECG) signal loss and artifacts, causing significant abnormalities in HR results. Following these exclusions, the final study T1DM group consisted of 15 participants, while the control group included 33 participants.

The University of Eastern Finland, in collaboration with the endocrinology and diabetology outpatient clinic at Kuopio University Hospital, was responsible for recruiting the participants. Recruitment efforts were made through local news outlets, associations, the university campus, and various social media platforms. For the T1DM group, the inclusion criteria were a diagnosis of T1DM without neuropathy, nephropathy, retinopathy, asthma, hypertension, or other chronic diseases. The criteria specified a duration of T1DM between 3 and 25 years, an age range of 18–50 years, and a body mass index (BMI) under 35 kg/m^2^. The control group was required not to have any chronic illnesses that necessitated medication, with the same age and BMI range as the T1DM group, and we aimed for an approximately 1:1 male‐to‐female ratio during recruitment for both groups.

The exclusion criteria for both groups included: (1) a history of coronary artery disease or heart attack, (2) amputation of a lower limb, (3) the presence of a pacemaker, (4) renal insufficiency, indicated by an estimated glomerular filtration rate (eGFR) below 60 mL/min or rapidly progressing microalbuminuria and edema, (5) nephrotic syndrome, (6) use of medications affecting the cardiovascular system, (7) severe asthma requiring ongoing medication, (8) current smoking habits, and (9) physical limitations preventing exercise.

All T1DM participants were on insulin therapy. Approximately half used multiple daily injections (MDI), while the other half used an insulin pump. One T1DM participant reported 2–3 hypoglycemic events in the previous 12 months prior to the test day. The participant stated these events did not affect testing. Two T1DM participants were on a mild cholesterol medication (rosuvastatin 5 mg).

### 2.2 Experimental protocols

The experiments were carried out in the Human Measurement and Analysis (HUMEA) lab at the University of Eastern Finland, with testing times scheduled from 7 a.m. to 2 p.m. Participants were instructed to consume a normal breakfast and a small lunch if necessary on the test day, and to refrain from consuming alcohol, caffeine, and tobacco products for 12 h, as well as to avoid intense physical activity for 24 h before undergoing the tests. Before the main tests, participants underwent a series of medical screenings, including a review of medical history, blood pressure measurement, lung function assessment via spirometry, and a fingertip blood sample was collected from T1DM participants to verify that blood glucose levels were within the range of 5–13.9 mmol/L. Blood glucose levels were measured before and after each test in accordance with applicable standards (Colberg et al., 2016). If the initial screenings revealed no health concerns, blood samples were drawn from the brachial vein and placed in ethylenediaminetetraacetic acid (EDTA) tubes for HbA_1c_ analysis. The HbA_1c_ levels were measured following the manufacturer's protocol using a cobas 8000 (c 702) analyzer (Hitachi High Technology, Tokyo, Japan). The use of bronchodilators was not allowed before or during the tests.

### 2.3 Self‐reported physical activity questionnaire

Participants were asked to report on their physical activities over the past 3 months, specifying the intensity, frequency, and volume. They were required to indicate how many days per week they engaged in each intensity level and the average duration in minutes per session. For example, light activities included walking and commuting, moderate activities involved mild breathlessness, such as brisk walking or participation in sports, and vigorous activities included significant breathlessness, such as high‐intensity interval training and competitive sports.

### 2.4 CPET protocol

CPET was conducted to investigate cardiopulmonary function during incremental exercise on a cycle ergometer (Ergoline Ergoselect 200 K, Ergoline GmbH, Bitz, Germany). The protocol began with a 3‐min resting period while the participant remained seated on the ergometer. For men, the exercise protocol started at an initial workload of 35 W, with increments of 35 W every 3 min. For women, the protocol started at 25 W, with increments of 25 W every 3 min. Sex‐specific workload increments were used because cycle ergometer work rate is an absolute external workload, and CPET protocols should be scaled to the participant's expected exercise capacity to support a fatigue‐limited exercise duration of approximately 8–12 min. This reduces the likelihood that exercise is terminated because of local muscular fatigue rather than cardiopulmonary endpoints (Balady et al., 2010). A forced expiratory maneuver was performed at the end of each workload increment throughout the test. Verbal encouragement was provided throughout the test to help participants achieve volitional peak effort. The test was terminated if the participant could not maintain a cadence above 60 rpm or achieved at least one of the following peak effort criteria: exceeding 95% of the predicted maximum HR, attaining a respiratory exchange ratio (RER) greater than 1.1, or exhibiting a plateau in oxygen uptake.

Throughout the exercise test, respiratory gases were analyzed using the breath‐by‐breath method with a Metamax 3b portable spiroergometry device (Cortex Biophysik GmbH, Germany). The spirometry system was calibrated with a 3000 mL calibration syringe (Medikro Calibration Syringe, Model M9474, Medikro, Kuopio, Finland). Cardiovascular function was assessed via impedance cardiography (Physioflow Enduro, Physioflow, France), which estimates SV from thoracic impedance changes and has been compared with direct Fick measurements at rest and during dynamic exercise (Charloux et al., 2000). Cardiac output (CO) was calculated from SV and HR, and cardiac index was calculated as CO divided by body surface area. Two channels of ECG were continuously recorded (Leads I and II) at a sampling frequency of 2000 Hz using the ME6000 biomonitor (Bittium, Oulu, Finland). Participants with T1DM had blood glucose monitored throughout the test using finger‐prick glucose measurements as required.

Oxygen pulse (O_2_ pulse) was calculated as V̇O_2_ divided by HR and was interpreted as an indirect gas‐exchange‐derived index related to SV, rather than as a direct measure of SV. According to the Fick principle, O_2_ pulse reflects the product of SV and arterial‐to‐venous oxygen difference and therefore also depends on peripheral oxygen extraction.

### 2.5 Determination of ventilatory thresholds

VTs were determined by a sports medicine physician using CPET software outputs and visual review of the ventilatory response patterns. The thresholds were analyzed from the CPET data using a combination of established criteria. For VT1 determination, a dual‐method approach was employed, involving an observed increase in the carbon dioxide production to oxygen uptake ratio (V̇CO_2_/V̇O_2_) slope, the modified V‐slope method, together with a nadir or turning point in the minute ventilation to oxygen uptake ratio (V̇E/V̇O_2_) equivalent curve (Weisman & Zeballos, 1994). For VT2 determination, the criteria included an increase in the minute ventilation to carbon dioxide production ratio (V̇E/V̇CO_2_) slope and a downward deflection in end‐tidal carbon dioxide partial pressure (PETCO_2_) (Carriere et al., 2019). RER was used as contextual support for threshold interpretation, but not as a standalone criterion. No formal secondary validation was performed.

### 2.6 Determination of peak exercise variables

All gas exchange data were averaged in 30‐s moving windows, and the highest 30‐s average recorded during the final completed workload stage was taken as the peak value (Hyrylä et al., 2025; Vogler et al., 2010). R‐R intervals obtained from the two‐lead ECG were converted to HR, smoothed with a 10‐s moving average, and the highest value within the same 30‐s epoch was designated as HR peak. Impedance cardiography signals were exported as ensemble averages of approximately 12 consecutive beats (Charloux et al., 2000); the largest ensemble average recorded within this identical 30‐s epoch was adopted as the cardiovascular peak.

### 2.7 Statistical analysis

All data were checked for normality using histograms, QQ‐plots, and the Shapiro–Wilk test (*n* < 50). Approximately half of the CPET and self‐reported physical activity variables were found to deviate significantly from a normal distribution (*p* < 0.05). For self‐reported physical activity variables, between‐group comparisons were performed using the Mann–Whitney *U* test, with statistical significance defined as *p* < 0.05. Between‐group median differences were estimated using the Hodges–Lehmann estimator with 95% confidence intervals (CIs) (Flechner & Tseng, 2011). Mann–Whitney *U* results were interpreted as tests of between‐group distributional differences, whereas Hodges–Lehmann estimates were used to describe the estimated median difference and its 95% CI. If the 95% CI included zero, the estimated median difference was interpreted as not statistically significant.

CPET variables measured repeatedly across exercise stages (rest, VT1, VT2, and peak) were analyzed using linear mixed‐effects models to account for within‐subject dependence and incomplete repeated measures. Group (T1DM vs. control), Stage, and Group × Stage were specified as fixed effects; subject was included as a random intercept, and Stage was treated as a categorical repeated factor within subject. The Group × Stage interaction therefore tested whether the stage‐wise CPET response differed between groups across rest, VT1, VT2, and peak exercise, rather than only comparing isolated peak values or rest‐to‐peak change scores. Models were estimated using restricted maximum likelihood (REML). Within‐subject correlation across stages was modeled using an autoregressive covariance structure of order 1 [AR(1)], selected based on model convergence and stability, as more complex structures resulted in convergence failures or non‐positive definite solutions. Type III tests of fixed effects with Satterthwaite‐adjusted degrees of freedom were used for inference.

Estimated marginal means with 95% CI were obtained for all Group × Stage combinations. As Group and Group × Stage effects were not significant for any CPET variable, no post hoc comparisons were reported. All analyses were conducted using IBM SPSS Statistics (Version 29.0.2.0 for Microsoft Windows, IBM Corporation, Armonk, New York).

## 3 RESULTS

### 3.1 Participants

Table 1 presents the characteristics of the study participants. The T1DM group consisted of 15 participants, while the control group included 33 participants. The groups were comparable in terms of sex, age, weight, height, and BMI, with no statistically significant differences observed between the T1DM and control groups for any of these characteristics. In the T1DM group, the average HbA_1c_ was 57.9 ± 7.5 mmol/mol (7.4 ± 0.7%), and the average duration of T1DM was 15 years, and all control participants had HbA_1c_ values within the normal reference range.

**TABLE 1:** Participant characteristics.

Variable	T1DM (*n* = 15)	Control (*n* = 33)	*p*
Women/men ratio	8:7	14:19	0.494^a^
Age (years)	32 ± 9	33 ± 7	0.896
Weight (kg)	74.0 ± 11.2	72.9 ± 11.4	0.752
Height (cm)	174.0 ± 8.5	174.5 ± 9.7	0.858
BMI (kg/m^2^)	24.3 ± 2.5	23.8 ± 2.4	0.525
HbA_1c_ (mmol/mol)	57.9 ± 7.5	31.8 ± 2.4	**<0.001**
HbA_1c_ (%)	7.5 ± 0.7	5.1 ± 0.2	**<0.001**
T1DM duration (years)	15 ± 7		

*Note*: Values are presented as mean ± SD. Bold: significant if *p* < 0.05.

Abbreviations: BMI, body mass index; HbA_1c_, glycated hemoglobin.

^a^ Chi‐squared test used for significance testing.

### 3.2 Self‐reported physical activity level results

Table 2 presents the self‐reported physical activity levels for the T1DM and control groups, including light‐intensity (LI), moderate‐intensity (MI), and high‐intensity (HI) physical activities. The data are presented in terms of frequency, volume (minutes per session), and total physical activity per week (hours). For LI frequency per week, the Mann–Whitney *U* test indicated a statistically significant between‐group distributional difference (*p* = 0.028). However, the Hodges–Lehmann estimated median difference was not statistically significant because its 95% CI included zero.

**TABLE 2:** Self‐reported physical activity level results.

Variable	T1DM (*n* = 15)	Control (*n* = 33)	Difference, 95% CI	*p*
LI frequency per week	3 (2; 5)	5 (3; 7)	2 (0, 3)	**0.028**
LI minutes a day	60 (40; 60)	45 (30; 60)	−10 (−25, 0)	0.224
MI frequency per week	2 (1.5; 4)	2 (2; 3)	0.5 (−0.5, 1.0)	0.328
MI minutes a day	60 (40; 90)	60 (30; 67.5)	0 (−30, 15)	0.553
HI frequency per week	1 (0; 2)	1 (0.35; 1.25)	0 (−0.5, 0.5)	0.608
HI minutes a day	30 (0; 60)	45 (15; 60)	0 (−15, 30)	0.709
Total per week (hours)	5.5 (4.9; 9.0)	7.0 (4.7; 10.6)	1.0 (−0.9, 3.0)	0.443

*Note*: Values are presented as median (25th; 75th). Median differences are presented as Hodges–Lehmann estimates with 95% CI. Bold: significant if *p* < 0.05.

### 3.3 CPET work rate and pulmonary function results

**FIGURE 1:**
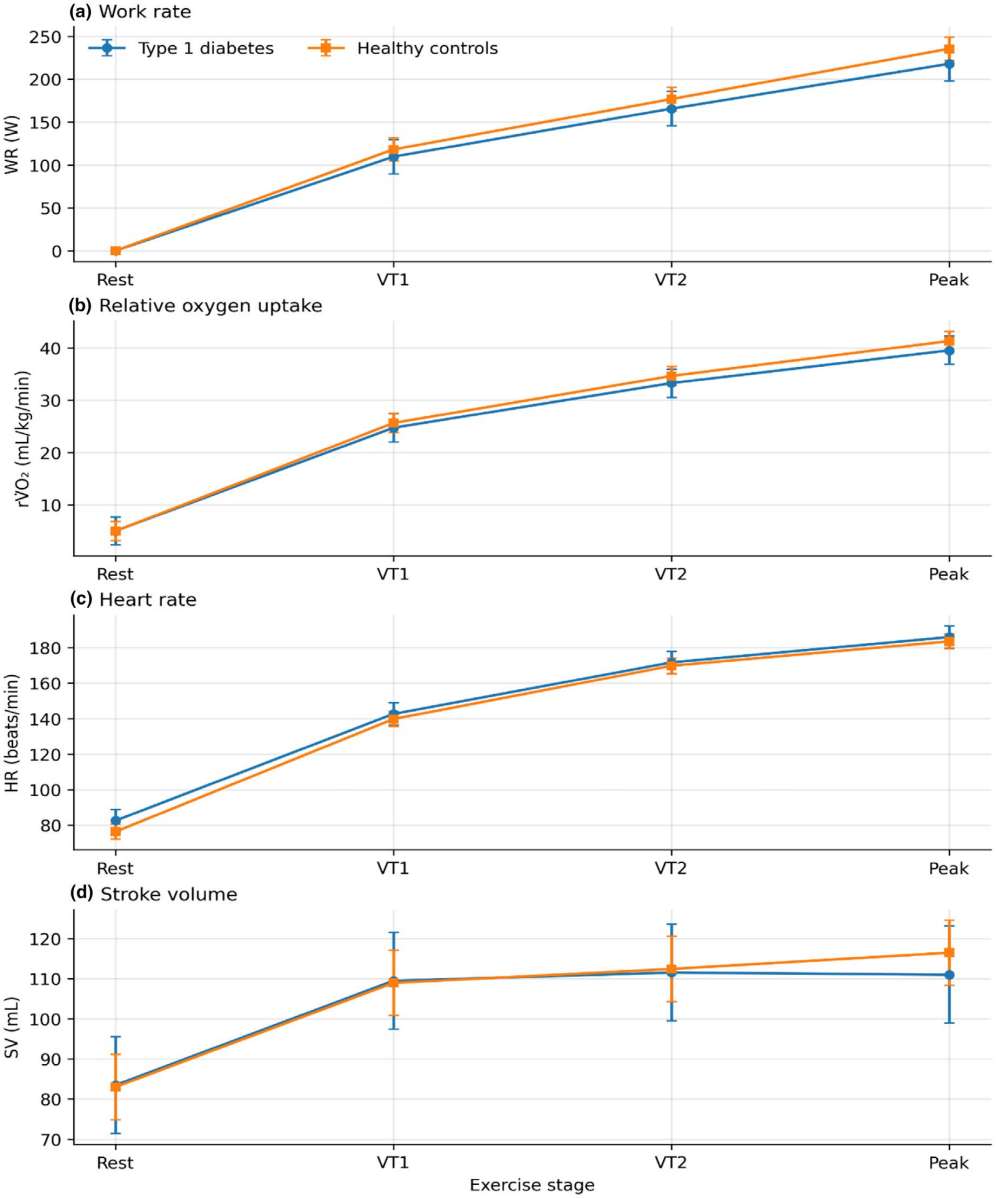
Stage‐wise work rate, relative oxygen uptake, heart rate, and stroke volume responses during CPET. Data are presented as estimated marginal means with 95% CI for T1DM and control at rest, VT1, VT2, and peak exercise. Panels show (a) work rate (WR), (b) relative oxygen uptake (rV̇O_2_), (c) heart rate (HR), and (d) stroke volume (SV).

Table 3 presents the CPET work rate (WR) and pulmonary function results for the T1DM and control groups at rest, VT1, VT2, and peak exercise. Figure 1a,b presents the stage‐wise profiles for WR and relative oxygen uptake (rV̇O_2_). Significant Stage effects were observed for all WR and pulmonary function variables. No significant Group or Group × Stage effects were detected for any variable, and none of these effects approached statistical significance.

**TABLE 3:** CPET work rate and pulmonary function results.

	T1DM (*n* = 15)	Control (*n* = 33)	*p*‐values for mixed models
WR (W)			
Rest	0 (fixed)	0 (fixed)	Group: 0.363
VT1	109.60 (89.48, 129.72)	118.09 (104.52, 131.66)	**Stage: <0.001**
VT2	165.67 (145.54, 185.79)	176.79 (163.22, 190.35)	Interaction: 0.484
Peak	218.00 (197.88, 238.12)	235.45 (221.89, 249.02)	
rV̇O_2_ (mL/kg/min)			
Rest	5.00 (2.31, 7.69)	4.97 (3.15, 6.79)	Group: 0.466
VT1	24.73 (22.04, 27.43)	25.64 (23.82, 27.45)	**Stage: <0.001**
VT2	33.27 (30.57, 35.96)	34.64 (32.82, 36.45)	Interaction: 0.625
Peak	39.53 (36.84, 42.23)	41.33 (39.52, 43.15)	
V̇O_2_ (L/min)			
Rest	0.37 (0.10, 0.63)	0.36 (0.18, 0.54)	Group: 0.749
VT1	1.83 (1.56, 2.09)	1.86 (1.69, 2.04)	**Stage: <0.001**
VT2	2.47 (2.21, 2.74)	2.52 (2.34, 2.70)	Interaction: 0.900
Peak	2.92 (2.66, 3.18)	3.01 (2.84, 3.19)	
O_2_ pulse (mL/beat)			
Rest	4.60 (2.89, 6.31)	4.97 (3.82, 6.12)	Group: 0.550
VT1	12.87 (11.16, 14.58)	13.39 (12.24, 14.55)	**Stage: <0.001**
VT2	14.33 (12.62, 16.04)	14.91 (13.76, 16.06)	Interaction: 0.921
Peak	15.67 (13.96, 17.38)	16.48 (15.33, 17.64)	
V̇E (L/min)			
Rest	12.15 (2.71, 21.58)	12.38 (6.02, 18.74)	Group: 0.544
VT1	46.45 (37.02, 55.89)	48.21 (41.85, 54.57)	**Stage: <0.001**
VT2	76.89 (67.45, 86.32)	78.67 (72.31, 85.03)	Interaction: 0.591
Peak	122.47 (113.04, 131.91)	129.93 (123.57, 136.29)	
VT (L)			
Rest	1.02 (0.75, 1.29)	1.04 (0.86, 1.22)	Group: 0.456
VT1	2.13 (1.87, 2.40)	2.21 (2.03, 2.39)	**Stage: <0.001**
VT2	2.41 (2.14, 2.68)	2.63 (2.45, 2.81)	Interaction: 0.496
Peak	2.46 (2.19, 2.73)	2.57 (2.39, 2.75)	
V̇E/V̇O_2_ (ratio)			
Rest	27.03 (24.99, 29.07)	27.75 (26.38, 29.13)	Group: 0.350
VT1	23.57 (21.53, 25.61)	24.07 (22.69, 25.44)	**Stage: <0.001**
VT2	28.80 (26.76, 30.84)	29.50 (28.12, 30.88)	Interaction: 0.937
Peak	39.39 (37.35, 41.43)	40.77 (39.39, 42.14)	
V̇E/V̇CO_2_ (ratio)			
Rest	30.69 (29.14, 32.25)	31.65 (30.60, 32.69)	Group: 0.329
VT1	25.48 (23.93, 27.03)	25.63 (24.59, 26.68)	**Stage: <0.001**
VT2	28.40 (26.85, 29.95)	28.84 (27.79, 29.89)	Interaction: 0.581
Peak	35.46 (33.91, 37.01)	36.85 (35.80, 37.89)	
V̇CO_2_ (L/min)			
Rest	0.32 (0.06, 0.59)	0.31 (0.13, 0.49)	Group: 0.743
VT1	1.70 (1.43, 1.96)	1.75 (1.57, 1.93)	**Stage: <0.001**
VT2	2.52 (2.25, 2.78)	2.57 (2.39, 2.75)	Interaction: 0.927
Peak	3.24 (2.97, 3.50)	3.32 (3.14, 3.50)	
PETCO_2_ (mmHg)			
Rest	34.93 (33.31, 36.56)	33.24 (32.15, 34.34)	Group: 0.256
VT1	42.93 (41.31, 44.56)	42.73 (41.63, 43.82)	**Stage: <0.001**
VT2	39.73 (38.11, 41.36)	38.82 (37.72, 39.91)	Interaction: 0.417
Peak	31.87 (30.24, 33.49)	30.88 (29.78, 31.97)	
RER (ratio)			
Rest	0.88 (0.85, 0.90)	0.86 (0.84, 0.88)	Group: 0.949
VT1	0.93 (0.90, 0.95)	0.94 (0.92, 0.96)	**Stage: <0.001**
VT2	1.01 (0.99, 1.04)	1.02 (1.00, 1.04)	Interaction: 0.588
Peak	1.11 (1.08, 1.14)	1.10 (1.09, 1.12)	
Breathing frequency (breaths/min)			
Rest	12.40 (9.69, 15.11)	12.97 (11.14, 14.80)	Group: 0.856
VT1	21.93 (19.23, 24.64)	22.06 (20.23, 23.89)	**Stage: <0.001**
VT2	32.07 (29.36, 34.77)	30.76 (28.93, 32.58)	Interaction: 0.437
Peak	49.87 (47.16, 52.57)	51.39 (49.57, 53.22)	

*Note*: Values are estimated marginal means with 95% CI. WR at rest is fixed at 0 W by protocol and is shown as 0 (fixed). *p*‐values are from Type III tests for Group, Stage, and Group × Stage. Bold: significant if *p* < 0.05.

Abbreviations: O_2_ pulse, oxygen pulse; PETCO_2_, end‐tidal carbon dioxide partial pressure; RER, respiratory exchange ratio; rV̇O_2_, relative oxygen uptake; V̇CO_2_, carbon dioxide production; V̇E, minute ventilation; V̇E/V̇CO_2_, ventilatory equivalent for carbon dioxide; V̇E/V̇O_2_, ventilatory equivalent for oxygen; V̇O_2_, oxygen uptake; VT, tidal volume; WR, work rate.

### 3.4 Cardiovascular function results

Table 4 presents the CPET cardiovascular function results for the T1DM and control groups at rest, VT1, VT2, and peak exercise. Figure 1c,d presents the stage‐wise profiles for HR and SV. Significant Stage effects were observed for all cardiovascular variables. HR increased progressively from rest to peak exercise in both groups. SV increased from rest to VT1, after which further changes were limited compared with the continued increases in HR, CO, and cardiac index. No significant Group or Group × Stage effects were detected for any cardiovascular variable, indicating that these stage‐wise patterns did not differ statistically between groups.

**TABLE 4:** CPET cardiovascular function results.

	T1DM (*n* = 15)	Control (*n* = 33)	*p*‐values for mixed models
HR (bpm)			
Rest	83 (76, 89)	76 (72, 81)	Group: 0.271
VT1	143 (136, 149)	140 (136, 144)	**Stage: <0.001**
VT2	172 (165, 178)	170 (166, 174)	Interaction: 0.687
Peak	186 (180, 192)	184 (179, 188)	
SV (mL)			
Rest	83.47 (71.38, 95.55)	83.00 (74.85, 91.15)	Group: 0.848
VT1	109.47 (97.38, 121.55)	108.94 (100.79, 117.09)	**Stage: <0.001**
VT2	111.53 (99.45, 123.62)	112.42 (104.28, 120.57)	Interaction: 0.288
Peak	111.00 (98.91, 123.09)	116.45 (108.31, 124.60)	
CO (L/min)			
Rest	6.92 (5.13, 8.71)	6.35 (5.14, 7.56)	Group: 0.899
VT1	15.56 (13.77, 17.35)	15.18 (13.97, 16.38)	**Stage: <0.001**
VT2	19.14 (17.35, 20.93)	19.02 (17.81, 20.22)	Interaction: 0.619
Peak	20.70 (18.91, 22.49)	21.31 (20.10, 22.52)	
Cardiac index (L/min/m^2^)			
Rest	3.73 (3.01, 4.46)	3.45 (2.96, 3.94)	Group: 0.928
VT1	8.22 (7.50, 8.95)	8.02 (7.53, 8.51)	**Stage: <0.001**
VT2	10.11 (9.38, 10.83)	10.06 (9.57, 10.55)	Interaction: 0.397
Peak	10.89 (10.17, 11.62)	11.30 (10.81, 11.79)	
EF (%)			
Rest	63.33 (59.50, 67.16)	63.42 (60.84, 66.01)	Group: 0.821
VT1	74.27 (70.44, 78.10)	72.55 (69.96, 75.13)	**Stage: <0.001**
VT2	73.53 (69.70, 77.36)	73.36 (70.78, 75.94)	Interaction: 0.608
Peak	71.73 (67.90, 75.56)	71.61 (69.02, 74.19)	
EDV (est.) (mL)			
Rest	130.07 (112.25, 147.89)	127.85 (115.83, 139.86)	Group: 0.938
VT1	149.20 (131.38, 167.02)	149.64 (137.62, 161.65)	**Stage: <0.001**
VT2	154.40 (136.58, 172.22)	153.76 (141.74, 165.77)	Interaction: 0.225
Peak	157.27 (139.45, 175.09)	162.94 (150.92, 174.95)	
SVR index (dyn·s·cm^−5^·m^2^)			
Rest	2083.13 (1963.23, 2203.03)	2048.03 (1967.19, 2128.87)	Group: 0.375
VT1	944.73 (824.83, 1064.63)	908.91 (828.07, 989.75)	**Stage: <0.001**
VT2	771.00 (651.10, 890.90)	720.61 (639.77, 801.44)	Interaction: 0.970
Peak	718.47 (598.57, 838.37)	648.36 (567.53, 729.20)	
LVET (ms)			
Rest	367.33 (352.80, 381.86)	361.67 (351.87, 371.46)	Group: 0.732
VT1	273.27 (258.74, 287.80)	275.67 (265.87, 285.46)	**Stage: <0.001**
VT2	241.87 (227.34, 256.40)	250.21 (240.42, 260.01)	Interaction: 0.610
Peak	219.53 (205.00, 234.06)	222.85 (213.05, 232.64)	

*Note*: Values are estimated marginal means with 95% CI. HR values are rounded to the nearest beat per minute. *p*‐values are from Type III tests for Group, Stage, and Group × Stage. Bold: significant if *p* < 0.05.

Abbreviations: CO, cardiac output; EDV (est.), estimated end‐diastolic volume; EF, ejection fraction; HR, heart rate; LVET, left ventricular ejection time; SV, stroke volume; SVR index, systemic vascular resistance index.

## 4 DISCUSSION

We investigated whether CPET responses were different between adults with uncomplicated T1DM and healthy controls matched for age, sex, height, weight, and BMI. For self‐reported physical activity, the Mann–Whitney *U* test indicated a statistically significant between‐group difference in the distribution of light‐intensity activity frequency, whereas the other variables did not differ significantly. However, the Hodges–Lehmann estimated median difference for light‐intensity activity frequency was not statistically significant because its 95% CI included zero. No statistically detectable Group effects or Group × Stage interactions were observed for work rate, pulmonary function, or cardiovascular function variables during CPET. These findings indicate that conventional CPET variables did not show statistically detectable between‐group differences under the present study conditions and sample size.

### 4.1 Self‐reported physical activity

Although the Mann–Whitney *U* test indicated a statistically significant between‐group difference in the distribution of light‐intensity activity frequency, the Hodges–Lehmann estimated median difference was not statistically significant because its 95% CI included zero. This finding should therefore be interpreted cautiously. The participants reported the weekly frequency of each activity intensity separately, and some reported more than one activity intensity on the same day. Therefore, the frequency variables were not restricted to 7 days per week and did not directly reflect the number of physically active days. As a result, the difference in light‐intensity activity frequency may partly reflect differences in how activity was classified or reported within a day rather than a true difference in overall physical activity. This interpretation is further supported by the absence of corresponding differences in total weekly physical activity or in the other activity variables. Nevertheless, self‐reported physical activity should be interpreted with caution, as participants' perceptions of their habitual activity may differ from objectively measured physical activity (Prince et al., 2008).

The physical activity profile of the present cohort should be considered when interpreting the CPET findings. Many adults with T1DM do not achieve recommended physical activity levels, and fear of hypoglycemia is a common barrier to regular physical activity (Brazeau et al., 2008; Matson et al., 2018; McCarthy et al., 2016). In the present study, participants were physically active and reported similar total weekly physical activity between groups. This may partly explain why conventional CPET responses did not differ statistically between the T1DM and control groups, but it also limits generalization to less active adults with T1DM.

Previous evidence also suggests that adults with uncomplicated T1DM can improve cardiorespiratory fitness in response to regular exercise. Rissanen et al. (2018) reported that a 1‐year unsupervised individualized exercise training intervention increased peak pulmonary oxygen uptake and peak O_2_ pulse in both adults with T1DM and healthy controls, with no significant Group × Time difference, despite no improvement in glycemic control in the T1DM group. Together, these findings suggest that the capacity to improve or maintain cardiorespiratory fitness through regular exercise may be retained in some adults with uncomplicated T1DM, although this should not be generalized to the broader T1DM population without caution.

### 4.2 Pulmonary function

Pulmonary function variables did not show statistically detectable Group effects or Group × Stage interactions across CPET, suggesting that marked ventilatory or gas‐exchange differences were not evident in this physically active and clinically selected uncomplicated T1DM cohort. However, previous comparative CPET studies have reported varying results regarding pulmonary function in T1DM. A study by Wilson et al. (2017) involving an uncomplicated T1DM group reported higher peak breathing frequency and nearsignificant differences in V̇O_2_max, maximal ventilation (VEmax) and workload in T1DM, findings that are absent in our study. Our stricter exclusion of complications, comorbidities and confounding medications may explain this discrepancy. Wilson et al. (2017) did not specify the frequency or intensity of participants' physical activity. In diabetes, frequent exercise interrupts prolonged sedentary periods, while high‐intensity sessions improve skeletal muscle oxidative capacity (Colberg et al., 2016). Short bouts of vigorous activity may also reduce the risk of hypoglycemic episodes (Tonoli et al., 2012). These exercise characteristics modulate cardiorespiratory adaptations and could therefore confound CPET comparisons. Furthermore, our cohort had mean BMI values within the normal‐weight range, in contrast to the overweight participants in the study by Wilson et al. (2017), based on BMI classification. Additionally, our T1DM group had lower HbA_1c_ levels than some previously studied cohorts. Earlier work suggested that metabolic control may influence physical fitness in insulin‐dependent type 1 diabetes (Poortmans et al., 1986), and recent evidence indicates that HbA1c and glycemic measures may be associated with graded exercise responses, including V̇O_2_max/V̇O_2_peak, peak power, and O_2_ pulse (Eckstein et al., 2022; McCarthy et al., 2023). However, HbA_1c_ alone appears insufficient to explain the heterogeneity of CPET responses in T1DM (Eckstein et al., 2022).

Evidence remains mixed. Jlali et al. (2023) reported normal resting lung function but reduced tidal volume and V̇O_2_max during CPET. Hypertension control was not specified, although participants' physical activity levels were similar to those in our study. Hyrylä et al. (2022) observed largely normal responses except for lower peak expiratory flow in adult men with well‐controlled T1DM. In a pooled analysis of observational cohorts that included microvascular complications, Eckstein et al. (2021) documented lower V̇O_2_max and reduced O_2_ pulse, while physical activity levels were similar between groups.

Taken together, these findings suggest that pulmonary responses to exercise in T1DM may depend on cohort characteristics and clinical profile rather than reflect a uniform impairment. In this context, our results suggest that conventional pulmonary and ventilatory CPET variables may not show detectable impairment in physically active adults with clinically selected uncomplicated T1DM, although larger cohorts are needed to confirm this interpretation.

### 4.3 Cardiovascular function

Cardiovascular variables did not show statistically detectable Group effects or Group × Stage interactions across CPET, suggesting that marked differences in conventional cardiovascular exercise responses were not evident under the present study conditions. Our results are in contrast with earlier studies. Eckstein et al. (2021) found a linear HR‐to‐performance curve in T1DM that ran consistently below the control curve, with blunted HR responses at VT1, VT2 and at peak effort. Moser et al. (2018) likewise observed a linear pattern in men with uncomplicated T1DM, whereas their control group showed the inverted pattern despite higher absolute HR values. Moser et al. (2018) attributed linearity to dysregulated cardiac contractility in T1DM, whereas Eckstein et al. (2021) argued linearity might not reflect reduced cardiac performance. Neither study assessed cardiovascular function beyond HR, leaving the contribution of SV dynamics unresolved. This is important because HR alone does not fully characterize cardiovascular adjustment to exercise. As exercise intensity increases, SV is shaped by ventricular filling time, ejection time, contractile function, and the transition toward the typical plateau pattern seen during incremental exercise. Without information on SV trajectories, it is therefore difficult to determine whether an altered HR‐to‐performance pattern reflects impaired central hemodynamics, compensatory regulation, or a physiologically preserved cardiovascular response. In this context, Haennel et al. (1993), who assessed both HR and SV, reported heterogeneous SV responses to incremental exercise in individuals with long‐term T1DM: seven participants failed to maintain an early rise in SV, showing a decline of more than 15%, eight displayed only a delayed increase, and five maintained a steady rise from rest to peak effort.

In the present study, SV increased from rest to VT1 and then showed a plateau‐like pattern from VT1 to peak exercise, whereas HR continued to increase. During these stages, left ventricular ejection time (LVET) shortens by ≈50 ms, indicating more rapid ejection of each stroke (Lance & Spodick, 1976). Although LVET shortened as exercise intensity rose and HR accelerated, SV was maintained, reproducing the classic SV plateau seen in incremental exercise (Stöhr et al., 2011). End‐diastolic volume (EDV) showed only modest, non‐directional fluctuations and ejection fraction (EF) fell slightly, consistent with prior research (Vieira et al., 2016). Thus, as exercise intensity and HR increase, there is less time for diastolic filling and ventricular ejection. Consequently, the heart compensates by contracting more forcefully to maintain SV, a pattern typically observed in recreationally active adults (Gledhill et al., 1994). The O_2_ pulse findings were consistent with the SV results, although O_2_ pulse should be interpreted cautiously because it reflects both SV and arterial‐to‐venous oxygen difference rather than SV alone (Chambers & Wisely, 2019).

These studies are not fully comparable with the present cohort. Moser et al. (2018) examined a small male‐only cohort with otherwise clinically similar uncomplicated T1DM, whereas Eckstein et al. (2021) analyzed a pooled, predominantly recent‐onset T1DM cohort with a median diabetes duration of 0.8 years, and Haennel et al. (1993) used careful clinical screening, but their cohort definition and cardiovascular phenotyping differed from those used in more recent uncomplicated T1DM studies. Thus, the discrepant cardiovascular findings across studies may reflect differences in cohort composition, diabetes phenotype, complication status, glycemic profile, and physical activity, rather than a uniform cardiovascular response pattern in T1DM.

Although no statistically detectable group differences were observed in conventional cardiovascular CPET variables, subtle autonomic or hemodynamic differences cannot be excluded. In adults with T1DM and cardiac autonomic neuropathy, graded exercise responses appear more clearly altered, including cardiopulmonary, sympathoadrenal, and metabolic impairments (McCarthy et al., 2026). This supports the interpretation that cardiac autonomic neuropathy can influence CPET responses and, if not accounted for, may confound comparisons of exercise responses in T1DM. However, in the present cohort, orthostatic testing demonstrated subtle increases in sympathetic activity and systolic blood pressure (Sorola et al., 2025), and post‐exercise analysis showed attenuated fast HR recovery after peak effort (Hyrylä et al., 2025). These findings suggest that subtle autonomic differences may be present in uncomplicated T1DM, but under the present sample size and group structure, they may not have shown statistically detectable differences in the measured conventional CPET variables.

### 4.4 Limitations

The small sample size, particularly in the T1DM group, is an important limitation and may have reduced statistical power to detect small or clinically subtle between‐group differences. This is especially relevant when interpreting nonsignificant findings, which should not be taken as evidence of physiological equivalence. However, modest sample sizes are common in CPET studies where participants are recruited and tested at one study site using detailed physiological measurements and carefully selected T1DM cohorts. In addition, related autonomic testing in the same cohort detected significant between‐group differences. Thus, the present findings should be interpreted as showing no statistically detectable differences in conventional CPET responses under the present study conditions, rather than as definitive evidence that no physiological differences exist. The present findings require confirmation in larger cohorts, preferably with prospective sample‐size planning and predefined clinically meaningful differences for key CPET outcomes.

The unequal group sizes should also be considered when interpreting the findings. Although the linear mixed‐effects model can accommodate unbalanced repeated‐measures data, the smaller T1DM group reduced the precision of between‐group estimates and may have limited the ability to detect small between‐group differences.

The clinically selected cohort also limits direct generalization to the broader heterogeneous T1DM population. However, this restriction was an intentional design feature, as the study aimed to reduce confounding from diabetes‐related complications, hypertension, smoking, cardiovascular medication, and major comorbidities. The findings should therefore be interpreted as applying to physically active adults with uncomplicated T1DM under the present CPET conditions, rather than to all individuals with T1DM.

The control group had a higher proportion of males, likely elevating median V̇O_2_peak and peak WR (Thomas et al., 2020). HR and V̇E/V̇CO_2_ are generally sex‐independent during CPET (Thomas et al., 2020), but men typically have higher SV. Nonetheless, SV was not significantly higher in the control group despite using sex‐specific loading protocols (25 W for women, 35 W for men).

We did not measure blood pressure during CPET, nor did we include recovery‐phase metrics. Orthostatic testing in the same cohort demonstrated subclinically elevated systolic blood pressure and heightened sympathetic activation (Sorola et al., 2025), and attenuated fast HR recovery after CPET (Hyrylä et al., 2025). Consequently, we cannot determine in this study whether standard CPET variables would capture these subtle cardiovascular and autonomic nervous system differences.

The self‐reported physical activity data should also be interpreted cautiously. The questionnaire assessed habitual physical activity over the previous 3 months and did not collect week‐by‐week activity data. In addition, participants reported the frequency of light‐, moderate‐, and high‐intensity activity separately, and some participants may have reported more than one activity intensity on the same day. Consequently, the frequency variables were not restricted to 7 days per week and did not directly represent the number of physically active days. The apparent between‐group difference in light‐intensity activity frequency may therefore partly reflect differences in how activity was classified or reported within a day, rather than a true difference in overall physical activity. This interpretation is supported by the absence of a statistically significant between‐group difference in total weekly physical activity. Future studies should use objective methods to assess physical activity rather than relying solely on subjective questionnaires.

Given existing evidence of subtle autonomic alterations in uncomplicated T1DM, future studies should examine heart rate variability (HRV) during CPET to determine whether more detailed autonomic responses emerge during exercise even when conventional CPET variables show no statistically detectable group differences. Continuous glucose monitoring was not assessed over the days or weeks preceding the testing day. Although blood glucose was checked on the test day according to protocol, preceding glycemic patterns and time in range could not be evaluated. This should be considered because longer‐term glycemic control has been associated with physiological responses during graded exercise testing in adults with T1DM, including V̇O_2_peak, peak power, and O_2_ pulse (McCarthy et al., 2023). Larger studies should therefore consider stratification or adjustment for time in range, HbA_1c_, training status, age, BMI, diabetes duration, and diabetes‐related complications.

## 5 CONCLUSION

In this physically active and clinically selected cohort, adults with uncomplicated T1DM showed no statistically detectable differences in conventional cardiopulmonary responses compared with healthy controls during CPET. These findings suggest that marked impairments in conventional CPET responses were not evident under the present study conditions. However, because blood pressure was not measured during CPET and autonomic nervous system responses during exercise were not evaluated, subtle subclinical hemodynamic or autonomic differences cannot be excluded, as suggested by orthostatic and post‐exercise recovery findings in the same cohort. Larger prospective studies are needed to confirm these findings within T1DM.

## AUTHOR CONTRIBUTIONS

**Samu Sorola:** Formal analysis; investigation; methodology; software; visualization; writing – original draft; writing – review and editing. **Timo Eronen:** Conceptualization; data curation; formal analysis; investigation; methodology; software; validation; writing – review and editing. **Vesa Hyrylä:** Conceptualization; formal analysis; methodology; software; validation; writing – review and editing. **Saana Kupari:** Data curation; writing – review and editing. **Mika Venojärvi:** Supervision; validation; writing – review and editing. **Heikki Tikkanen:** Conceptualization; methodology; project administration; resources; supervision; validation; writing – review and editing. **Mika Tarvainen:** Conceptualization; funding acquisition; methodology; project administration; resources; software; supervision; validation; writing – review and editing. **Harri Lindholm:** Investigation; methodology; supervision; validation; writing – review and editing.

## FUNDING INFORMATION

This study was (funded through an EFSD award) supported by EFSD/JDRF/Lilly and by the Finnish Diabetes Foundation. European Foundation for the Study of Diabetes, 96108, Mika Tarvainen.

## CONFLICT OF INTEREST STATEMENT

None.

## ETHICS STATEMENT

The DIAMES study was approved by the Ethics Committee of the Northern Savo Hospital District, Kuopio, Finland (reference number 409/2019). All participants provided written informed consent before participation. The study was conducted in accordance with the Declaration of Helsinki, good clinical practice, and the General Data Protection Regulation.

## Data Availability

The data are not publicly available because of participant privacy and ethical restrictions. Upon reasonable request to the corresponding author, de‐identified data may be partially made available, subject to applicable ethical and data protection requirements.
