# Correction to “Physically active adults with uncomplicated type 1 diabetes exhibit normal cardiopulmonary exercise test responses versus healthy controls”

**DOI:** 10.14814/phy2.71084

**Published:** 2026-09-02

Sorola, S. , Eronen, T. , Hyrylä, V. , Kupari, S. , Venojärvi, M. , Tikkanen, H. , Tarvainen, M. , & Lindholm, H. (2026). Physically active adults with uncomplicated type 1 diabetes exhibit normal cardiopulmonary exercise test responses versus healthy controls. Physiological Reports, 14, e70997. 10.14814/phy2.70997.42374909 PMC13316121

In paragraph 1 of Section 2.4 (“CPET protocol”), the sentence “A forced expiratory maneuver was performed at the end of each workload increment throughout the test” was incorrect. This should have read: “An inspiratory capacity maneuver was performed at the end of each workload increment throughout the test.”

This was an error in the written description of the experimental procedure and does not affect the collected data, statistical analyses, results, interpretation, or conclusions of the article.

We apologize for this error.

